# Adaptive Control of Pressure Difference in Abrasive Flow Machining for Inner Hole with High Depth/Diameter Ratio

**DOI:** 10.3390/ma17246123

**Published:** 2024-12-14

**Authors:** Yaojie Cai, Panyu Qian, Donghui Wen, Guixiang Jin, Mingsheng Jin, Qiaoling Yuan, Li Zhang

**Affiliations:** 1 Key Laboratory of Special Purpose Equipment and Advanced Processing Technology, Ministry of Education and Zhejiang Province, Zhejiang University of Technology, Hangzhou 310023, China; caiyj@zjut.edu.cn (Y.C.); 211123020008@zjut.edu.cn (P.Q.); jinguixiang@confirmware.com (G.J.); jms611@163.com (M.J.); yuanql@zjut.edu.cn (Q.Y.); zhangli@zjut.edu.cn (L.Z.); 2 College of Mechanical Engineering, Zhejiang University of Technology, Hangzhou 310023, China; 3 Logistics Equipment Development Department, Hangzhou ConfirmWare Technology Co., Ltd., Hangzhou 310052, China

**Keywords:** inner hole, high depth/diameter ratio, abrasive flow machining, flow field pressure difference, adaptive regulation

## Abstract

To address the issue of uneven pressure distribution in the abrasive flow field within the inner hole of components with a large depth/diameter ratio, an adaptive control strategy for regulating flow field pressure difference is proposed in this paper. The strategy was based on the effects of pressure fluctuations at the abrasive flow inlet and outlet on pressure distribution patterns and pressure changes within the inner hole flow field, as derived from numerical simulations. An adaptive control fixture was also designed, enabling dynamic adjustments to the fixture gap, which significantly reduced the pressure difference in the flow field. Experimental results demonstrated that the surface texture uniformity of the inner hole was greatly improved after adaptive control. The non-uniformity in surface roughness after control was 59.1% lower compared to pre-control conditions, indicating improved machining consistency. Furthermore, the maximum reduction in surface roughness increased from 1.146 μm to 1.844 μm, and processing efficiency was notably enhanced.

## 1. Introduction

The equipment manufacturing industry is the pillar of the real economy. Increasing demands for equipment and product quality in fields such as national defense, military, transportation, and aerospace have led to stricter surface quality requirements for inner holes in components with high depth/diameter ratios. In defense and military applications, high surface quality is essential for the stable and reliable performance of weapon systems, as even minor defects can impact accuracy and combat effectiveness. Especially in transportation, the surface quality of inner holes in key components, such as high-speed trains and aero-engines, is critical to both safety and operational efficiency. These components are often subjected to extremely harsh operating conditions, and a smooth inner surface can enhance both high-temperature resistance and high-pressure performance. By eliminating small cracks and stress concentration points on the surface, fatigue strength and wear resistance can be significantly improved, thereby extending the service life of equipment. Moreover, a high-quality surface finish helps reduce friction and minimize unnecessary energy loss, thereby improving overall efficiency. Therefore, manufacturing enterprises must continuously innovate processing technologies and elevate standards to achieve optimal surface quality in inner holes with high aspect ratios [1,2,3].

**Figure 1:**
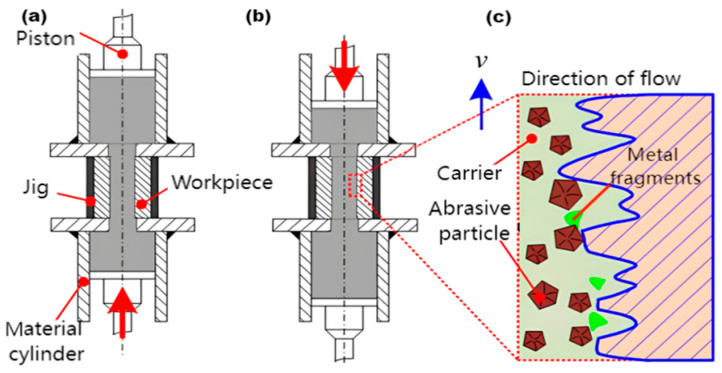
The principle of abrasive flow machining. (**a**) Abrasive moves from bottom to top. (**b**) Abrasive moves from top to bottom. (**c**) Material removal mechanism of abrasive flow machining.

Abrasive flow machining (AFM) offers flexibility, profiling capabilities, and high accessibility in fluid polishing. The machined surface is always subjected to abrasive action, which can achieve efficient material removal, resulting in a high-quality surface [4]. The processing principle is illustrated in Figure 1. AFM squeezes semi-solid, viscoelastic, and abrasive media into the channel under high pressure to process the inner surface. Micro-cutting and micro-ploughing represent the primary material removal mechanisms in abrasive flow machining [5]. Abrasive water jets (AWJs) also utilize abrasive media for processing, with material removal occurring via plastic deformation, erosion, and shear mechanisms caused by the impact of abrasive particles on the workpiece [6]. AFM and AWJs exhibit significant similarities in the influence of process parameters, such as abrasive particle size, type, hardness, and workpiece characteristics on surface quality, yet they differ markedly in processing parameters, material removal mechanisms, and theoretical models [5,7,8]. Both AWJs and AFM have become popular choices in the field of surface processing due to their unique characteristics. However, these technologies do have certain limitations. For example, jet delays of AWJs can influence process accuracy. Therefore, an appropriate trade-off between processing efficiency and nozzle lifespan must be made. Furthermore, vibrations during machining can adversely affect surface quality and operational safety [6,9,10]. The edge effect, wall effect, pressure difference effect, and other factors in the AFM process also significantly impact overall machining performance [11,12].

Among them, the pressure difference refers to the non-uniform pressure distribution within the abrasive flow field and the variation of pressure differences along the flow path, which arise from changes in the flow channel interface and pressure losses during the viscoelastic abrasive flow process. This results in variations in the surface roughness and texture of the workpiece along the abrasive flow direction. Peng et al. [13] investigated the influence of wall slip behavior on the inhomogeneity of AFM processing. Based on capillary flow and the Mooney method modified by Bagley, a prediction model, including a nonlinear slip equation, was developed, providing a basis for understanding the complex correlation between processing inhomogeneity and wall slip behavior. The high consistency of experimental and prediction results demonstrated that this model offers superior accuracy, practicality, and applicability compared to the linear slip model. Wang et al. [14] explored the influence of key processes on flow field changes through numerical simulations, identified the impact of process parameters on machining quality, and minimized variations in surface roughness and texture of the machined surface. Addressing the inhomogeneity observed in traditional AFM of polygonal holes, Chen et al. [15] carried out numerical simulations with spiral channels and square holes as the research objects, finding that the machining efficiency of the spiral channel is superior to that of the square channel. Peta [16] analyzed surface complexity in electrical discharge machining and found that the complexity of surface microgeometry and roughness significantly influences the wettability and frictional properties of liquids on the surface. At an appropriate scale, surface roughness and microgeometry complexity can balance the wettability and frictional properties of the surface. Li et al. [17] used the servo valve shell as the research subject, adjusted the back pressure by changing the outlet diameter of the valve body, and numerically simulated the solid–liquid two-phase abrasive flow machining process to examine the effects of varying back pressure outlet diameters on dynamic pressure and wall shear force. The results show that when the back pressure outlet diameter is 3.3 mm, the dynamic pressure and wall shear force are maximized, resulting in the highest processing efficiency and optimal surface quality.

Currently, most research primarily focuses on analyzing overall pressure differences, with limited investigation into uneven processing caused by local pressure variations. This study investigated the impact of pressure difference effects on machining hole parts with a depth/diameter ratio of 10:1. The workpiece was divided into four axial units, and a control strategy for pressure difference homogenization was developed, aiming at regulating both local and overall pressure differences within the workpiece. The feasibility of this control strategy was then verified through experiments, providing theoretical insights and technical support for the precise and efficient polishing of cylindrical inner holes with high depth/diameter ratios.

## 2. Experimental Study

### 2.1. Experimental Equipment and Method

**Figure 2:**
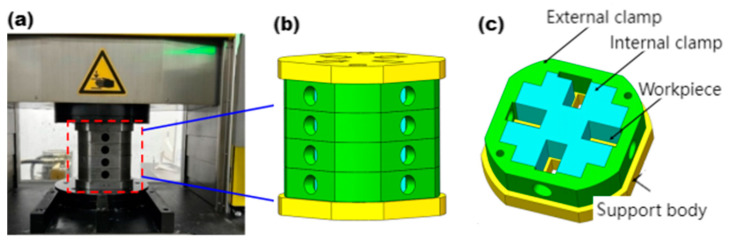
Processing platform and fixture structure. (**a**) Abrasive flow extrusion equipment. (**b**) Integral fixture. (**c**) Integral fixture section view.

The experiment utilized the Easy Flow 200 abrasive flow extrusion equipment, as illustrated in Figure 2. The entire fixture comprises an inner clamp, an outer clamp, and a support body. The combined height of the inner and outer clamps is 200 mm, while each support body has a height of 30 mm. To facilitate installation, the inner and outer clamps are divided into four layers, with four workpieces featuring different processing methods installed in each layer. The fixture was constructed from 45 steel. To enable an intuitive analysis of the fixture’s internal structure, a three-dimensional cross-sectional diagram of the entire assembly is presented in Figure 2c. Each layer of clamps accommodates four workpieces, which are maintained in a vertical orientation and subjected to the same processing method. To prevent the workpieces from falling into the cylinder, a support body with a square hole corresponding to the clamp is positioned at both the upper and lower sections of the clamp. During the processing, we use an abrasive particle size of 240 mesh.

**Figure 3:**
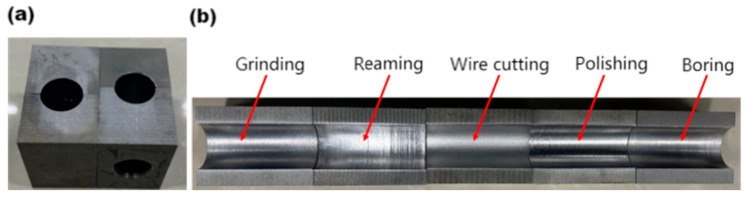
(**a**) The workpiece. (**b**) Hole surfaces under different processing methods.

The material of the workpiece to be processed is 42CrMo. The dimensions of the workpiece are 59 mm × 35 mm × 50 mm, featuring a through hole on the end face with a diameter (D0) of 20 mm. To facilitate the assessment of surface morphology and roughness of the cylindrical inner hole before and after processing, the workpiece was cut halfway at a 1/3 eccentricity, as illustrated in Figure 3a. The surfaces of various inner holes were processed using five machining techniques: reaming, boring, grinding, wire cutting, and polishing, which were selected as the subjects for conducting a uniformity test of abrasive flow pressure differences. The physical representation is illustrated in Figure 3b, while the specific machining processes applied to the workpiece surface are detailed in Table 1.

Various machined surfaces were employed as variables for AFM, with the experimental steps meticulously designed and organized to yield more accurate and reliable data. The specific steps are as follows: (1) The ultrasonic cleaning machine was set to a frequency of 60 Hz, with a cleaning duration of 3 min. (2) Four workpieces in each processing mode were numbered, with the designations of unit 1, unit 2, unit 3, and unit 4 engraved using a diamond pen, facilitating easy distinction when they were placed in the fixture and measured. (3) The 2D surface texture, 3D surface profile, and surface roughness of the four workpieces for each processing method were measured based on the number assigned to the center point of the inner hole surface. (4) The workpiece was installed. (5) The relevant parameters were set on the control panel. (6) Upon completion of the processing, the workpieces were removed, and the 2D surface texture, 3D surface profile, and surface roughness of the workpieces for each machining method were measured again. (7) The test data were processed and analyzed.

### 2.2. Experimental Results

#### 2.2.1. Analysis of Surface Roughness Before and After Processing

**Figure 4:**
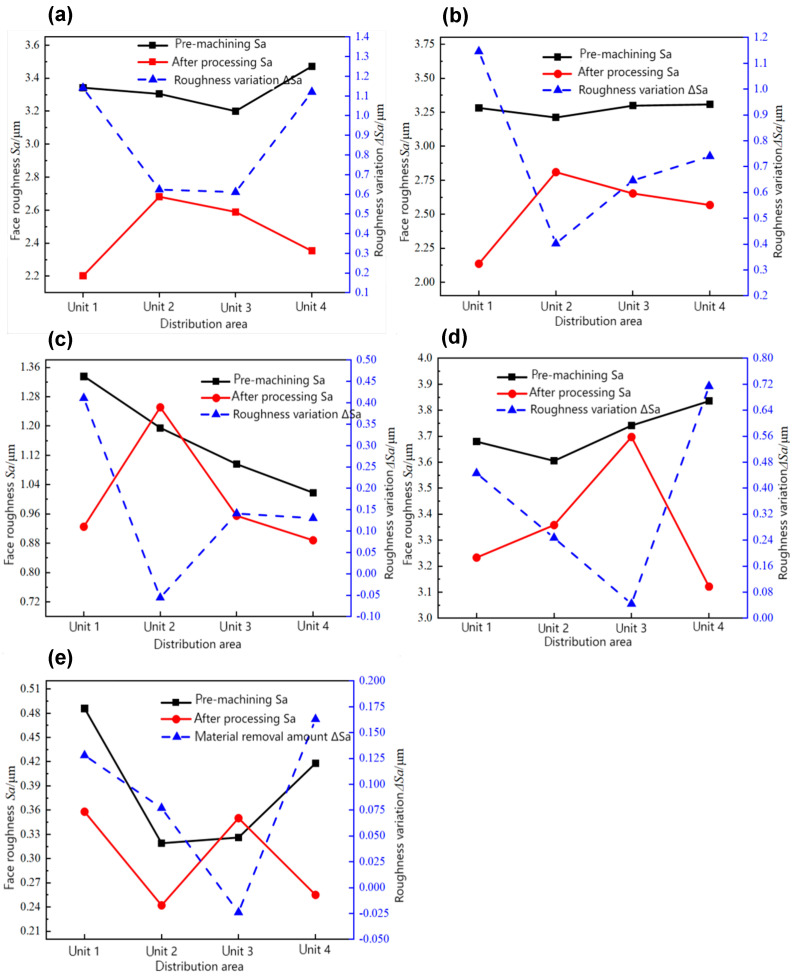
Comparison of material removal before and after abrasive flow machining on surfaces processed by different processes: (**a**) reaming machining, (**b**) boring machining, (**c**) grinding processing, (**d**) wire cutting processing, and (**e**) polishing processing.

The surface roughness data for the inner holes of different units subjected to various processing methods, both before and after processing, were fitted and analyzed. As illustrated in Figure 4, different initial processing surfaces yield varying processing effects. Materials with higher initial surface roughness exhibit a relatively higher removal rate. However, due to the excessive roughness of the surface after wire cutting, the 240 abrasive particle size fails to achieve significant material removal. In contrast, the surface quality following grinding and polishing is satisfactory, but the low initial surface roughness limits further improvements in processing. Moreover, in certain smoother areas, surface roughness may increase due to the reprocessing effect of abrasives. Furthermore, analysis indicates that material removal in units 1 and 4 is more pronounced than in units 2 and 3.

#### 2.2.2. Analysis of Surface Uniformity Before and After Processing

To intuitively illustrate the difference in material removal on the workpiece surface before and after abrasive flow processing, we utilized the non-uniformity coefficient (δ). By defining the ratio of surface roughness amplitude $Sa_{\max}-Sa_{\min}$ to average surface roughness $Sa_{m}$, that is, $\delta =(Sa_{\max}-Sa_{\min})/Sa_{m}$, this study evaluates the fluctuation of surface roughness in the four units before and after processing, thus determining the impact of abrasive flow processing on surface uniformity.

**Figure 5:**
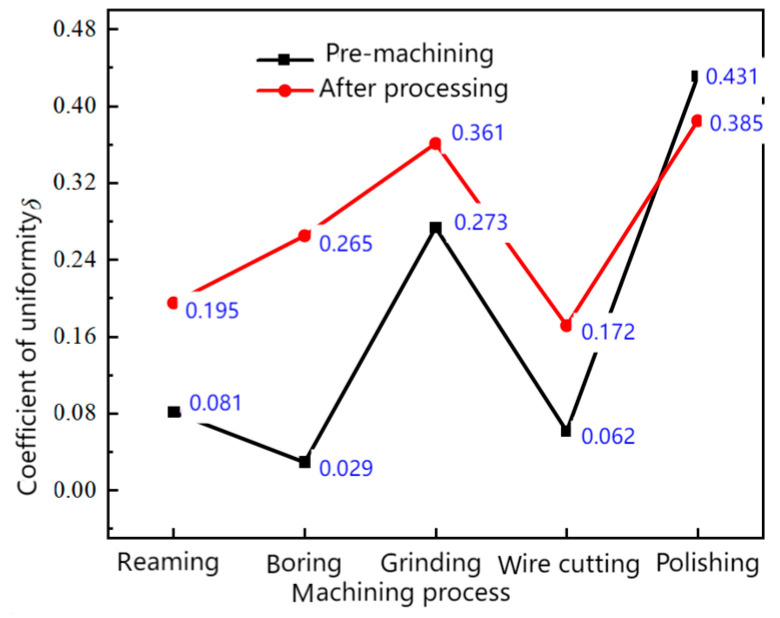
Uniformity comparison before and after abrasive flow machining for surfaces processed by different processes.

Figure 5 indicates that the surface uniformity of the polished workpiece is enhanced following AFM, potentially due to two reasons. Firstly, the comparison of material removal before and after machining, as shown in Figure 4e, reveals that the initial surface roughness of units 1 and 4 after polishing is slightly higher than that of units 2 and 3. Given that higher surface roughness correlates with improved machining effects, units 1 and 4 exhibit superior machining results compared to units 2 and 3. Additionally, the surface roughness of the four units displays less fluctuation, indicating improved uniformity. Secondly, the use of white light interference measurement, which assesses a range of 50 μm in the central area of the workpiece, leads to differences in measurement points before and after processing, resulting in variations in roughness measurements. Conversely, the surface uniformity of workpieces processed by other methods deteriorates, as shown in Figure 4a–d. When the initial surface roughness of the four units shows minimal variation, units 1 and 4 exhibit greater changes compared to units 2 and 3, leading to increased fluctuation in surface roughness and, consequently, poorer uniformity. Notably, the inner hole processed by boring shows the most significant change. Subsequent research will focus on the inner hole processed by boring as the primary subject.

## 3. Numerical Simulation

### 3.1. Models and Parameters

**Figure 6:**
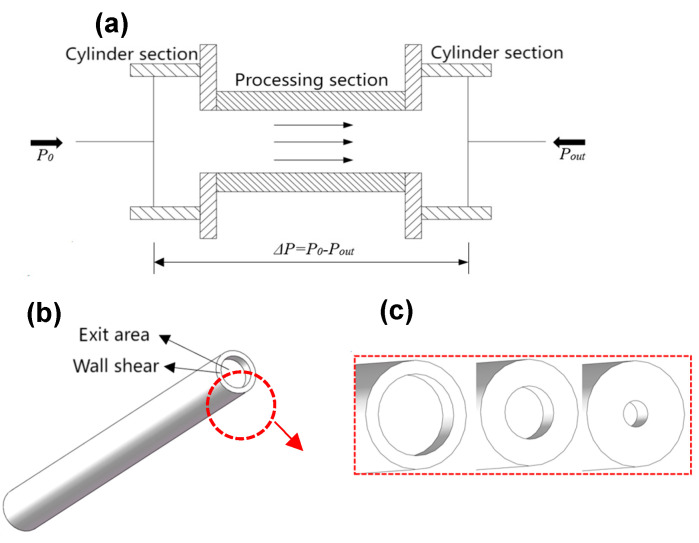
Variations in inlet and outlet pressure differences: (**a**) flow field pattern, (**b**) flow field model, and (**c**) different outlet areas.

Analysis from the previous section indicates that the surface of the inner hole has improved following AFM, with varying material removal effects across different workpieces. Notably, the workpiece processed by boring exhibits the most significant unevenness and will be the focus of subsequent research. The primary forces involved in AFM are positive pressure and axial shear force. This section will numerically simulate the impact of varying inlet and outlet pressure differences on the pressure distribution within the flow field, as well as the corresponding pressure changes in each unit. Utilizing the back pressure method, the flow field model was designed to stabilize the numerical range of inlet and outlet pressure differences with the objective of maximizing axial pressure while minimizing pressure differentials. Figure 6a illustrates that by applying back pressure at the outlet, the overall pressure difference in the flow field is expressed as ΔP = P0 − Pout. The flow field model is further enhanced by constructing it around the core circular hole section, as depicted in Figure 6b,c. Back pressure is adjusted by varying the outlet area size, allowing for the determination of the corresponding pressure difference through repeated numerical simulations.

The meshing function in the computational fluid dynamics (Fluent2022R1) software was utilized. Given that the shapes and sizes of the circular hole section, support section, and cylinder section differ significantly, the connections were refined to enhance the accuracy of the simulation, thereby better reflecting the actual physical phenomena. Considering that the circular hole section was the primary focus of this study, the mesh was further refined in this region. Additionally, to more accurately simulate and capture the interaction between the fluid or structural objects and their surrounding environment, a boundary layer was incorporated into the circular hole section, thereby enhancing the accuracy and reliability of the numerical simulation.

The abrasive consists of a two-phase incompressible fluid composed of silicon carbide (SiC) particles and polymer. It was assumed that the abrasive particles are spherical, with specific parameters detailed in Table 2. Consequently, a pressure-based solver utilizing the pressure method was employed, and the mixture model was selected for the multiphase flow analysis. When fluid flows through a circular pipe, the Reynolds number serves as a critical indicator for distinguishing between laminar, transitional, and turbulent flow. This study estimates the maximum Reynolds number Re=ρvdμ≤2300 under given simulation conditions, leading to the selection of the laminar flow model. Given that the test environment is a closed space, the energy equation is activated. The wall of the circular hole is defined as a static, non-slip, convective heat exchange boundary with a heat exchange coefficient of K = 20 W/(m^2^·K) to simulate natural convection heat transfer in contact with air.

### 3.2. Computational Results

Fluent2022R1 software was employed to model variations in the flow field within the cylindrical inner hole under different inlet and outlet pressure differences. The inlet pressure P0 = 1 MPa, the workpiece diameter D0 = 20 mm, and the workpiece length L = 200 mm were specified. The inlet and outlet pressure differences ΔP were established at 1, 0.76, 0.52, 0.28, and 0.04 MPa, respectively.

**Figure 7:**
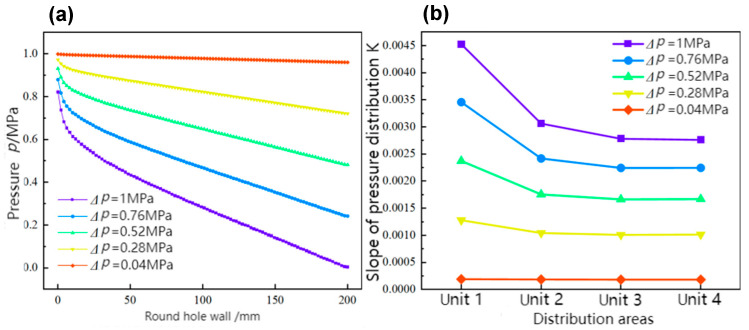
Effects of inlet and outlet pressure differences: (**a**) pressure distribution along the circular hole wall, and (**b**) pressure differential changes across each unit.

As illustrated in Figure 7a, the effects of varying pressure differentials on the pressure distribution along the wall of the circular hole are presented. It demonstrates that as the pressure difference decreases, the pressure at each point on the wall of the circular hole gradually increases, indicating that under conditions of reduced pressure differentials, the pressure distribution within the flow field tends to become more uniform. Furthermore, the pressure distribution curve gradually approaches a horizontal line, suggesting that the pressure difference between the inlet and outlet enhances axial positive pressure, resulting in a more uniform pressure change in the axial direction.

A further analysis of the variations in pressure differences for each unit, as illustrated in Figure 7b, presents the pressure differential change curves under different inlet and outlet pressure conditions. To investigate the influence of each parameter on the pressure difference of each unit during AFM, the slope K of each unit’s pressure distribution curve is analyzed. P50 is defined as the pressure at the end of unit 1, while P0 is defined as the pressure at the beginning of unit 1, representing the ratio of pressure differential/flow length of $K_{i}=\frac{\Delta P_{i}}{x_{i}}=\frac{P_{50}-P_{0}}{50}$. The influence of each parameter on the changes in pressure differentials across different units is examined in relation to this value. Given the substantial pressure differential in the inlet area, it is essential to initiate calculations from a distance of 10 mm to mitigate the impact of excessive pressure drop in the inlet area on the pressure difference changes throughout unit 1. Additionally, analysis of the non-uniformity coefficient δ indicates that when the inlet and outlet pressure difference is 1 MPa, the pressure difference in unit 1 exhibits significant changes, gradually decreasing along the flow direction. As the pressure difference between the inlet and outlet decreases, the pressure differences of the four units progressively decline, approaching a straight line. At this point, the uniformity of the pressure difference is optimal, as shown in Figure 7b, suggesting that the axial shear effect across the entire machining area tends to become consistent under these conditions. However, due to the minimal change in pressure difference, the abrasive flow rate is considerably slow, resulting in a diminished axial shear effect and consequently weakening the polishing outcome. Therefore, the pressure difference should not be excessively low. Considering processing efficiency, the optimal inlet and outlet pressure difference is determined to be 0.28 MPa.

## 4. Regulation Research

### 4.1. Regulatory Strategy

**Figure 8:**
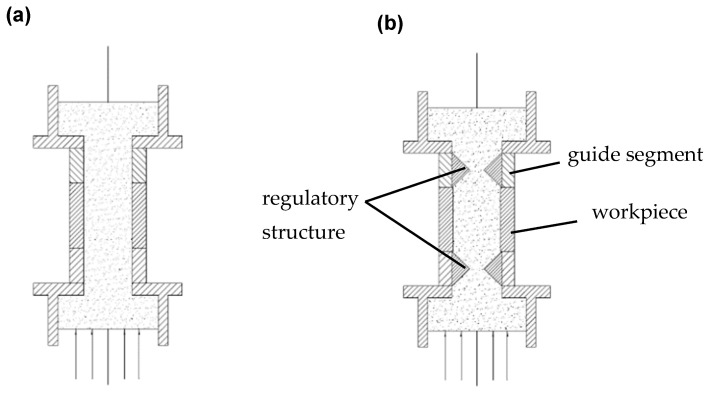
The regulation strategy: (**a**) traditional AFM and (**b**) adaptive motion.

For the round hole workpiece exhibiting a high depth/diameter ratio, the surface quality after processing is inconsistent, which can be attributed to the effects of pressure differentials. Based on the aforementioned numerical simulation results, an adaptive pressure difference control strategy is proposed to mitigate the impact of pressure differentials to the greatest extent possible. The specific implementation scheme is as follows: The control structure is installed both above and below the four-layer fixture. During the upward movement of the abrasive, the lower control structure adjusts the opening size based on the preset inlet pressure, while the upper control structure dynamically adjusts according to the pressure difference. By analyzing the collected pressure data and comparing it to a predetermined threshold, the opening and closing of the control structure are managed in real time. Conversely, when the abrasives move downward, the system operates to consistently maintain the pressure difference in the flow channel at a constant value, as shown in Figure 8.

**Figure 9:**
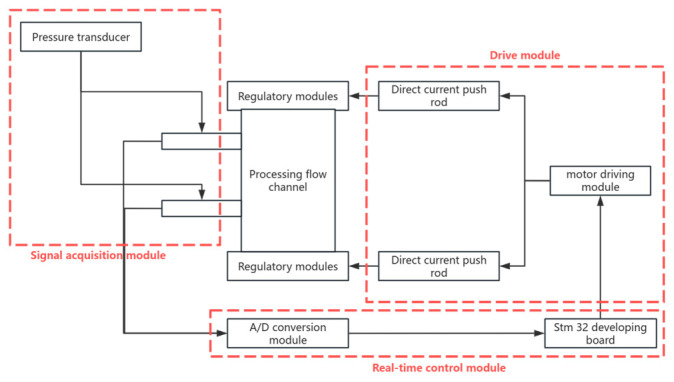
Schematic of the adaptive differential pressure control system.

According to the principles of the adaptive pressure difference control strategy described above, the overall implementation functions as a closed-loop feedback control process. Pressure signals at the inlet and outlet of the flow channel were collected, and the analog signals were converted into digital signals through the AD conversion module. The corresponding program then regulates the telescopic movement of the electric push rod to adjust the control structure and the abrasive outlet area, thereby achieving stable control of the pressure difference. The system structure is illustrated in Figure 9, which comprises a signal acquisition module, a real-time control module, and a drive module.

(1) Signal acquisition module

The signal acquisition module is a crucial component of the adaptive differential pressure control system, responsible for monitoring the inlet and outlet pressure signals of the flow channel. The workflow includes the following steps: The sensor transmits the analog signal to the module, which preprocesses and converts it into a digital signal via the analog-to-digital (A/D) converter for transmission to the real-time control module. This digital signal serves as the feedback closed-loop input, reflecting the real-time state of the pressure. The module facilitates the system’s acquisition of fluid pressure information, enables effective monitoring and control of the pressure difference, and provides essential input data.

(2) Real-time control module

**Figure 10:**
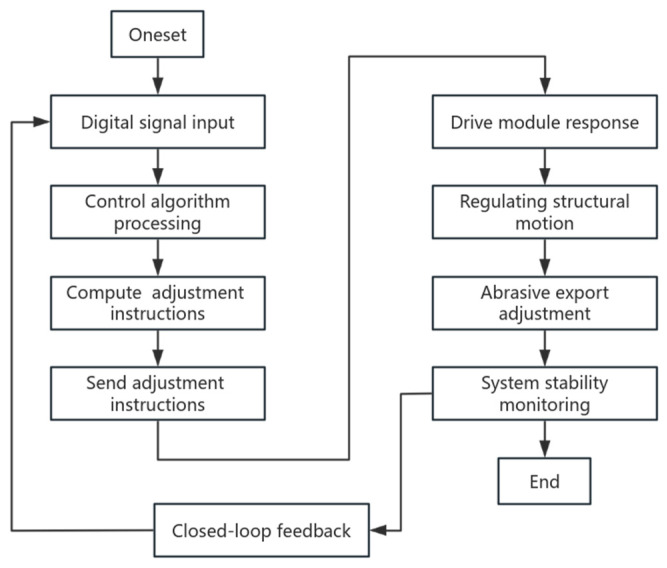
Real-time control flowchart.

The real-time control module serves as the core component of the adaptive pressure difference control system, responsible for processing pressure information from the signal acquisition module and dynamically adjusting the abrasive outlet area using algorithms. Figure 10 illustrates the module’s workflow. The digital signal obtained from the signal acquisition module reflects the real-time pressure at the inlet and outlet of the flow channel. The control algorithm processes this signal and sends adjustment instructions to the drive module, which governs the action of the electric push rod. The drive module controls the movement of the electric push rod and adjusts the abrasive outlet area to meet the pressure difference requirements. By continuously receiving new data, closed-loop feedback is established through ongoing calculation and adjustment, ensuring system adaptability and the maintenance of the pressure difference. The module enables timely monitoring and response to unstable factors, thereby ensuring system stability. Through the operation of the control module, the adaptive pressure difference control system can rapidly and accurately respond to pressure changes in the fluid channel, achieving precise control over the drive module.

(3) Drive module

The drive module is a critical component of the adaptive differential pressure control system, comprising the motor driver, electric push rod, and execution structure. It is responsible for controlling the extension of the electric push rod and applying force to the control structure, thereby adjusting the abrasive outlet area. The primary function of the drive module is to ensure that the motor driver supplies the appropriate current and voltage to the motor based on the voltage signal. Additionally, it controls the forward and reverse rotation of the motor to facilitate the telescopic movement of the electric push rod.

### 4.2. Structural Design

**Figure 11:**
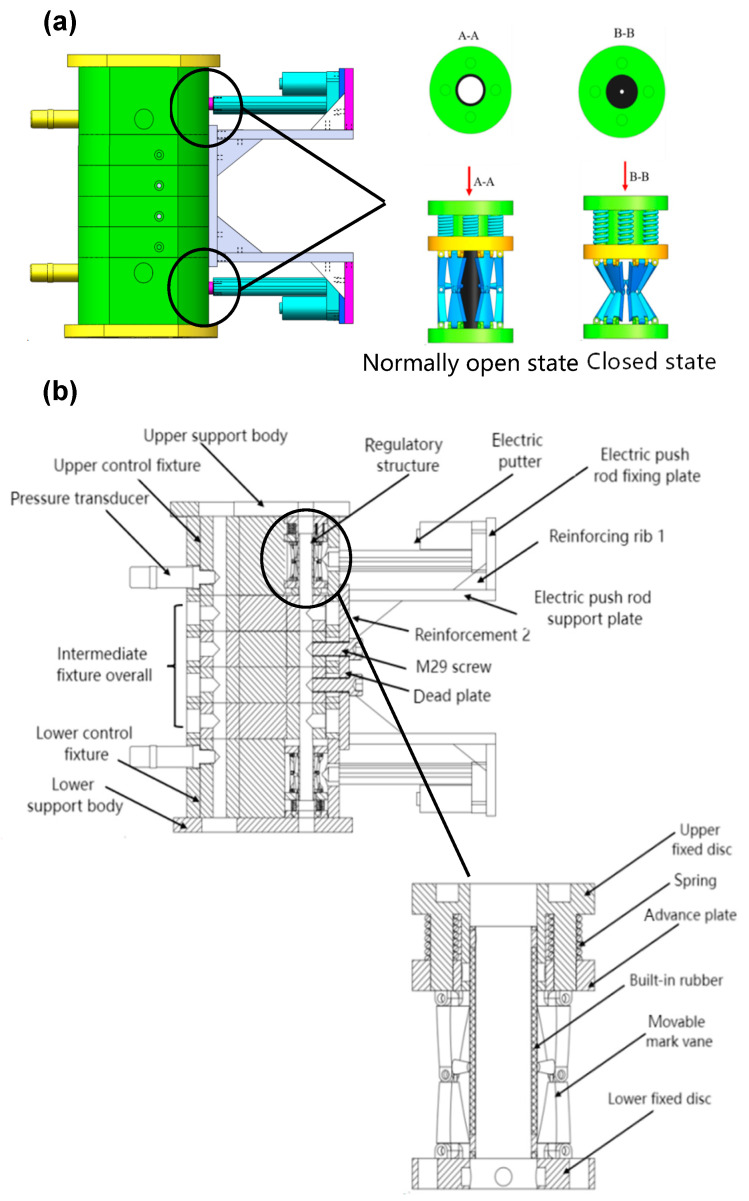
Overall structure diagram: (**a**) three-dimensional model and control structure opening/closing schematic and (**b**) two-dimensional sectional view.

The overall device structure is shown in Figure 11. To optimize space utilization, the primary component of the signal acquisition module, the pressure sensor, was installed on the opposite side of the processing flow channel, maintaining alignment with the processing flow. To prevent abrasive leakage, the threaded holes were sealed with screws. In the processing section, the threaded holes of workpiece units 2 and 3 were utilized to secure the connecting plate, while the threaded holes of units 1 and 4 were treated similarly to the opposite flow channel.

The electric push rod support plate and the electric push rod fixed plate were interconnected using screws. To mitigate the risk of deformation due to excessive force, a reinforcing rib (rib 1) was designed between the support plate and the fixed plate. Additionally, a second reinforcing rib (rib 2) was incorporated between the electric push rod support plate and the electric push rod fixed plate to enhance the overall strength of the device. The drive module, primarily facilitated by the electric push rod, was connected to the fixed plate via the push rod bracket secured with screws. If the cross-sectional change of the flow channel relies solely on the telescopic movement of the push rod, the flow field’s motion will be significantly altered, potentially affecting processing efficacy. Furthermore, to streamline installation, the new component materials incorporated into the original foundation clamp were made from 6061 aluminum alloy.

**Figure 12:**
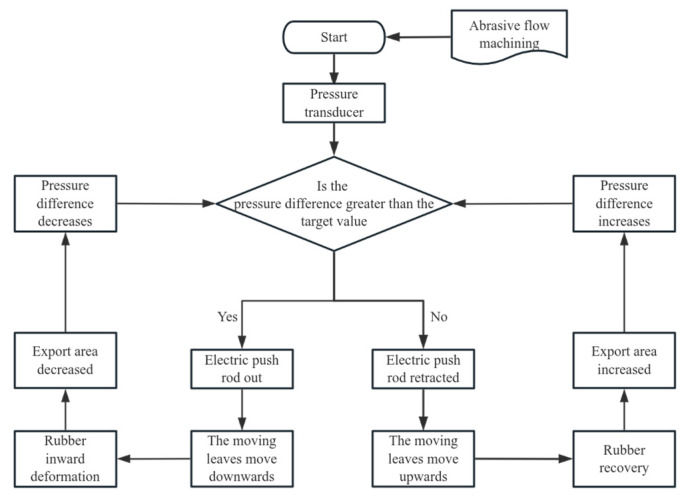
The working flowchart of the experimental structure.

The internal control structure was designed in an hourglass configuration, as illustrated in Figure 11b. This internal assembly consists of upper and lower fixed plates, an intermediate moving structure, a tensile spring, and a built-in rubber component. The intermediate moving structure includes both a moving blade and a moving plate. The upper and lower fixed plates are securely attached to the support plate and the intermediate fixture using screws. The upper fixing plate incorporates a guide shaft. The tensile spring is welded to both the upper fixing plate and the movable plate, while the built-in rubber component is affixed to the inner wall of the guide shaft with screws. The external drive module exerts force on the moving blade, causing it to move downward and apply extrusion pressure to the built-in rubber, thereby altering the outlet area of the flow channel. Upon release of the force, the tensile spring facilitates the return of the moving structure to its initial position, and the built-in rubber regains its original shape due to its inherent elasticity. This process completes the regulation of the outlet area of the processing flow channel. The opening and closing states are depicted in Figure 11a. The working flowchart of the experimental structure is shown in Figure 12.

### 4.3. Experimental Verification

**Figure 13:**
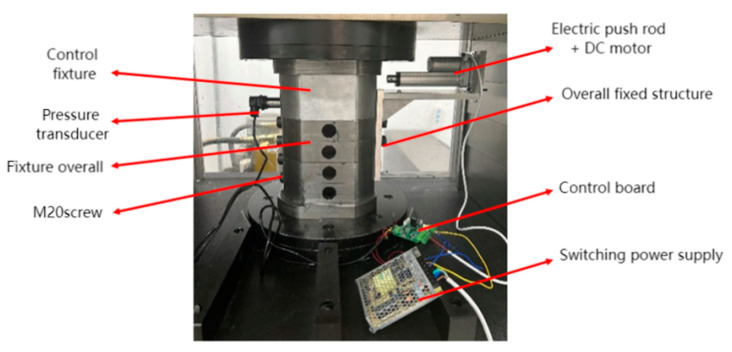
Test platform.

The control test platform is depicted in Figure 13. Based on the previously established control principles, a model has been designed to create the test platform, which facilitates two-way processing of the entire flow channel. Utilizing the construction device and control system, the test was developed with specific steps outlined as follows: (1) The installation, cleaning, and preliminary preparation of the entire fixture and workpiece were conducted according to the steps outlined in Section 2.1. (2) Install the sensors and wire the control system accordingly. (3) The number of machining cycles was configured to 35 on the control panel. During the upward movement of the abrasive, the control system was activated. Conversely, it remained inactive when the abrasive moved downward. (4) After completing 35 operations, the orientation of the workpiece containing the four built-in units was adjusted, and the procedure described in (3) was repeated to achieve 35 cycles of bidirectional abrasive flow processing. (5) Following the cleaning of the four units of the workpiece, the surface morphology and surface roughness were measured and compared to the pre-processing values. (6) Data analysis was conducted using Excel and Origin.

**Figure 14:**
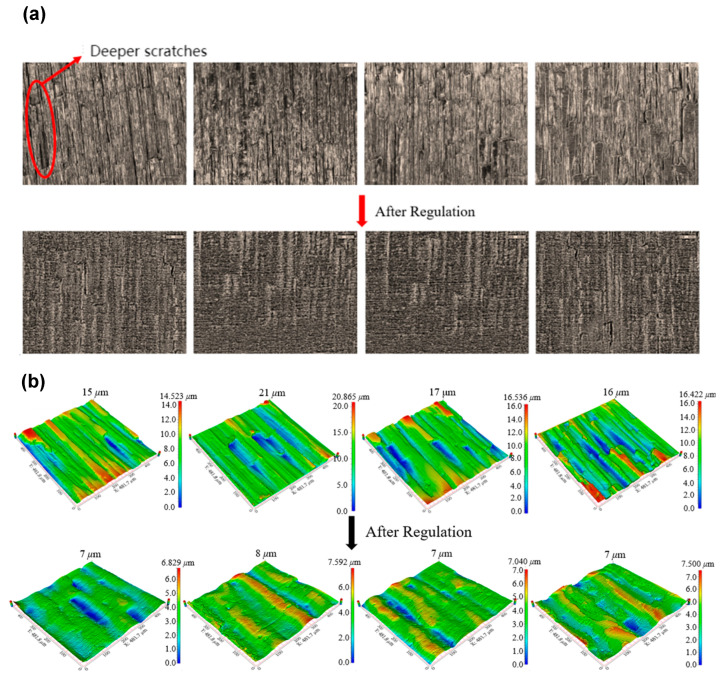
(**a**) Comparison of 2D surface texture before and after regulation and (**b**) comparison of 3D surface profiles before and after regulation.

Based on the previous numerical simulation results, the effect of pressure differentials can be effectively mitigated by adjusting the pressure difference between the inlet and outlet. This section presents an experimental analysis conducted in accordance with the proposed adaptive pressure difference control strategy. In the experiment, the inlet–outlet pressure differential was set to 0.28 MPa, the inlet pressure was maintained at 1 MPa, and the number of machining cycles was established at 35. The 2D surface texture and 3D surface profile post-processing results are displayed in Figure 14a,b; from left to right, these correspond to unit 1, unit 2, unit 3, and unit 4. It is evident that the scratches have been largely eliminated, with only a few deep scratches remaining, which are difficult to remove. Overall, the surface quality has been significantly improved. Regarding roughness peaks, the maximum roughness peak reduction in traditional AFM is 6 μm. However, after regulation, this reduction increases to 13 μm, while the roughness peak of the four units is reduced to approximately 7 μm, thereby significantly enhancing machining consistency.

Further analysis of surface roughness and uniformity before and after processing, as presented in Table 3, reveals that the non-uniformity of the machined surface roughness after regulation is 59.1% lower than that observed prior to regulation. This finding indicates that the processing effects across different units are largely consistent following regulation, resulting in enhanced stability of surface quality. Additionally, the variation in roughness has improved, reflecting a significant increase in the efficiency of abrasive flow machining.

## 5. Conclusions

For workpieces with a high depth/diameter ratio, traditional abrasive flow machining exhibits significant pressure difference effects, leading to poor surface uniformity in the machined workpiece. Existing research shows several limitations in addressing pressure difference uniformity. In this paper, an adaptive differential pressure control strategy was proposed, and its feasibility was validated through experimental processing. The main conclusions were as follows:

1. The surfaces of inner holes with a depth/diameter ratio of 10:1 were subjected to five machining processes—reaming, boring, grinding, wire cutting, and polishing. Abrasive flow machining was then performed, and surface roughness and roughness non-uniformity were used as evaluation metrics. The results indicate that surface quality non-uniformity after machining is significantly evident.
2. Numerical simulations were conducted to analyze pressure difference uniformity and establish the control range. Flow fields for various inlet and outlet pressure differentials were constructed and analyzed. The analysis shows that as the inlet–outlet pressure differential increases, the pressure at each point along the inner wall of the circular hole gradually rises, while the pressure differential in the inlet region diminishes. This suggests that increasing the inlet–outlet pressure differential enhances the machining effect while mitigating the over-thrusting effect in the inlet area. Furthermore, an increase in the pressure differential improved the uniformity of pressure differences across different units. However, the pressure differential should not exceed a certain threshold, as excessive differentials could reduce the axial shear effect, thereby affecting processing efficiency.
3. An adaptive pressure difference control strategy was proposed to effectively manage the pressure difference uniformity range, yielding positive results. Experimental findings demonstrated that the strategy effectively mitigated the pressure difference effect. When the inlet–outlet pressure difference (ΔP) was 0.28 MPa, the surface integrity of the four workpiece units was significantly improved after processing, with most surface scratches eliminated and roughness non-uniformity reduced by 59.1% compared to pre-regulation levels. Thus, the influence of the pressure difference effect is suppressed. At the same time, the efficiency and applicability of abrasive flow machining are improved, and high-quality precision polishing of cylindrical inner holes with a high depth/diameter ratio is realized.

## Figures and Tables

**Table 1:** Different surface processing methods and processes for inner holes.

Processing Methods	Machining Process
Reaming	The Φ19.85 mm after wire cutting is processed, and then the Φ20 mm reamer is processed at a speed of 150 rpm
Boring	Rough boring F200, speed 1000 rpmFine boring F50, speed 1000 rpm
Grinding	Φ19.8 mm after wire cutting, internal grinding speed 6000 rpm, workpiece speed 500 rpm
Wire cutting	Φ0.18 mm molybdenum wire, middle wire cutting
Polishing	After boring with a wool wheel at a speed of 2000 rpm

**Table 2:** Material parameters of abrasives.

Species	Parameter
Class	Green silicon carbide
Grain size	240# ^1^
Viscosity grade	B1
Mass fraction	25%
Density	1340 kg/m^3^

^1^ The symbol # refers to the number of holes per inch of the sieve. 240 # means 240 holes per inch.

**Table 3:** Surface roughness and non-uniformity coefficient of workpiece before and after machining.

Condition	Surface Roughness (μm)				δ (μm)
	Unit 1	Unit 2	Unit 3	Unit 4	
Pre-machining	3.281	3.211	3.298	3.307	0.005
Unregulated	2.135	2.809	2.652	2.567	0.265
After regulation	2.061	1.965	1.849	1.946	0.108

## Data Availability

The original contributions presented in this study are included in the article. Further inquiries can be directed to the corresponding author.
